# Different Sensitivity Levels of the Photosynthetic Apparatus in *Zea mays* L. and *Sorghum bicolor* L. under Salt Stress

**DOI:** 10.3390/plants10071469

**Published:** 2021-07-17

**Authors:** Martin A. Stefanov, Georgi D. Rashkov, Ekaterina K. Yotsova, Preslava B. Borisova, Anelia G. Dobrikova, Emilia L. Apostolova

**Affiliations:** Institute of Biophysics and Biomedical Engineering, Bulgarian Academy of Sciences, Acad. Georgi Bonchev Street, Block 21, 1113 Sofia, Bulgaria; martin_12.1989@abv.bg (M.A.S.); megajorko@abv.bg (G.D.R.); ekaterina_yotsova@abv.bg (E.K.Y.); preslavab12345@gmail.com (P.B.B.); aneli@bio21.bas.bg (A.G.D.)

**Keywords:** NaCl treatment, OJIP test, PAM chlorophyll fluorescence, photosynthesis, pigment composition, stress markers

## Abstract

The impacts of different NaCl concentrations (0–250 mM) on the photosynthesis of new hybrid lines of maize (*Zea mays* L. Kerala) and sorghum (*Sorghum bicolor* L. Shamal) were investigated. Salt-induced changes in the functions of photosynthetic apparatus were assessed using chlorophyll *a* fluorescence (PAM and OJIP test) and P_700_ photooxidation. Greater differences between the studied species in response to salinization were observed at 150 mM and 200 mM NaCl. The data revealed the stronger influence of maize in comparison to sorghum on the amount of closed PSII centers (1-qp) and their efficiency (Φ_exc_), as well as on the effective quantum yield of the photochemical energy conversion of PSII (Φ_PSII_). Changes in the effective antenna size of PSII (ABS/RC), the electron flux per active reaction center (REo/RC) and the electron transport flux further Q_A_ (ETo/RC) were also registered. These changes in primary PSII photochemistry influenced the electron transport rate (ETR) and photosynthetic rate (parameter R_Fd_), with the impacts being stronger in maize than sorghum. Moreover, the lowering of the electron transport rate from Q_A_ to the PSI end electron acceptors (REo/RC) and the probability of their reduction (φRo) altered the PSI photochemical activity, which influenced photooxidation of P_700_ and its decay kinetics. The pigment content and stress markers of oxidative damage were also determined. The data revealed a better salt tolerance of sorghum than maize, associated with the structural alterations in the photosynthetic membranes and the stimulation of the cyclic electron flow around PSI at higher NaCl concentrations. The relationships between the decreased pigment content, increased levels of stress markers and different inhibition levels of the function of both photosystems are discussed.

## 1. Introduction

Increases of soil salinity are a major environmental problem worldwide, affecting about 30% of irrigated land and 6% of the total land [1]. Ivushkin et al. [2] divided different soils according to their electrical conductivity (EC) into certain classes: non-saline (<2 mS/cm), slightly saline (2–4 mS/cm), moderately saline (4–8 mS/cm), very saline (8–16 mS/cm), and highly saline (>16 mS/cm). Salinity is one of the abiotic stress factors causing restriction of plant growth and crop productivity as a result of alterations in plant metabolism and enzyme activities [3,4,5,6,7].

Salt stress involves both ionic and osmotic stresses, which influence the process of photosynthesis, depending on the strength and duration of the stress, as well as the salt sensitivity of the plants [3,4,8,9,10,11]. According to the biphasic model for the growth responses of plants to high salt concentrations, the osmotic stress (osmotic deficit dominates) during the first phase and the ion toxicity during the second phase (ion effect dominates) cause the reduced growth in cereals [4,12]. It has been shown that salinity enhances the content of reactive oxygen species (ROS) in plant cells as a result of ion toxicity [13]. Under normal plant growth conditions, ROS act as signaling molecules during acclimatization to environmental stress factors, although excessive amounts of ROS in plants can lead to oxidation of pigments, lipids, proteins and nucleic acids [14]. Significant structural and functional changes in the photosynthetic machinery under salt stress connected with changes in the thylakoid membrane ultrastructure and composition were also established [14,15,16,17,18]. Salinity affects the photosystem II (PSII) complex more than the photosystem I (PSI) complex [13]. Moreover, the PSII complex is considered to be the most sensitive site of the photosynthetic apparatus under abiotic stress [19,20,21,22]. Previous investigations have revealed different effects of salt stress on PSII, depending on the salt tolerance of the plant species [8,15,18]. Changes have been shown in both the donor and acceptor sides of PSII, as well as changes in the PSII heterogeneity with respect to the antenna size [23,24]. The high salt concentrations influence the photosynthetic electron transport chain and decrease the photosynthetic activity [13,16,25]. Moreover, the influence of salt stress has been shown on the interaction between Q_A_ and plastoquinone (PQ), as well as on PSI antenna size [26,27]. The effects of high salt concentrations on the photosynthetic pigments also vary between different plant species. Previous studies have shown that in salt-tolerant species, the contents of chlorophylls and carotenoids increase, while in salt-sensitive species their amounts decrease [18,21,28,29,30].

In this study, experiments were conducted on maize (*Zea mays* L.) and sorghum (*Sorghum bicolor* L.), which are high-energy crops that are widely used [31,32]. Maize is the third most important cereal crop, growing under a wide spectrum of soil and climatic conditions [33]. Additionally, maize is considered moderately salt-sensitive [34]. Sorghum is the fifth most produced cereal globally [35] and is characterized as moderately salt-tolerant [36] and as a potential crop for moderately saline areas [37].

Keeping in mind the different impacts of salt stress on the functions of the PSII complex [8,15,18], which is considered to be the most sensitive site of the photosynthetic apparatus under abiotic stress [19,20,21,22], we hypothesize that changes in the primary photochemistry of PSII are related to plant tolerance to salinity. Our study examines the effects of different concentrations of NaCl (50 mM, 150 mM, 200 mM and 250 mM NaCl) on the function of the photosynthetic apparatus (PSII photochemistry and photooxidation of P_700_), the leaf pigment content and the markers of oxidative stress of two new hybrid lines of maize (*Zea mays* L. Kerala) and sorghum (*Sorghum bicolor* L. Shamal). Data show the differences as well as the similarities in the salt stress responses of maize and sorghum and provide additional information that will enrich our knowledge of the tolerance and adaptation mechanisms of these crop plants to increased amounts of salt in the soils.

## 2. Results

### 2.1. Photosynthetic Pigments

The pigment composition measurements showed that the amounts of chlorophylls (Chl) and carotenoids (Car) in control plants (untreated plants) were higher in maize compared to those of the sorghum (Table 1). The treatment with NaCl led to a reduction in the photosynthetic pigments, with greater effects at concentrations of 150 mM NaCl and higher. The decreases in Chl content after treatment with 150 mM and 200 mM NaCl were more pronounced in maize (37–58%) than in sorghum (28–47%). The smallest NaCl concentration (50 mM) induced a decrease of 12% in the content of total Chl and Car only in maize. The salt treatment also led to increases in the ratios Chl *a*/*b* and Car/Chl, as the ratio Chl *a*/*b* was greater in sorghum than in maize when treated with the same NaCl concentrations (Table 1).

### 2.2. Oxidative Stress Markers

**Figure 1:**
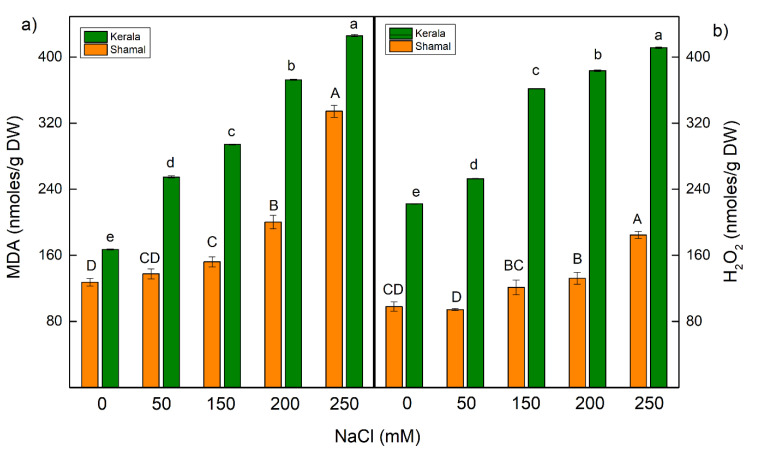
Effects of different NaCl concentrations on the content of MDA (**a**) and H_2_O_2_ (**b**) in leaves of sorghum (*Sorghum bicolor* L. Shamal) and maize (*Zea mays* L. Kerala). Data are given per dry weight (DW). Mean values (± SE) were calculated from 8 independent measurements. Different letters indicate significant differences for the respective parameters at *p* < 0.05 (capitals for sorghum and lowercase for maize).

The hydrogen peroxide (H_2_O_2_) contents in the leaves of control plants, as well as the increase of its amount after salt treatment, were higher in maize than in sorghum samples for all studied NaCl concentrations (Figure 1). In sorghum, salt-induced H_2_O_2_ accumulation was observed with treatment at 150 mM NaCl and above, while in maize H_2_O_2_ accumulation was observed even at 50 mM NaCl. Significant differences in the increases of H_2_O_2_ content between the two studied species were registered after treatment with 150 mM and 200 mM NaCl; for sorghum this increase was by 24–35%, while for maize it was by 63–72%.

The level of the lipid peroxidation (MDA content) corresponds to the oxidative damage of membranes (Figure 1). Similar to H_2_O_2_, the salt-induced changes in MDA amounts for both studied species were registered. In maize, increase in MDA were found at all studied NaCl concentrations; even at the lowest concentration (50 mM NaCl) this increase was by 52%. Data also revealed smaller increases in MDA content at all applied NaCl concentrations for sorghum in comparison to maize. In general, the increases in both H_2_O_2_ and MDA contents in maize were higher than in sorghum, regardless of the salt concentration (Figure 1).

### 2.3. Electrolyte Leakage

**Figure 2:**
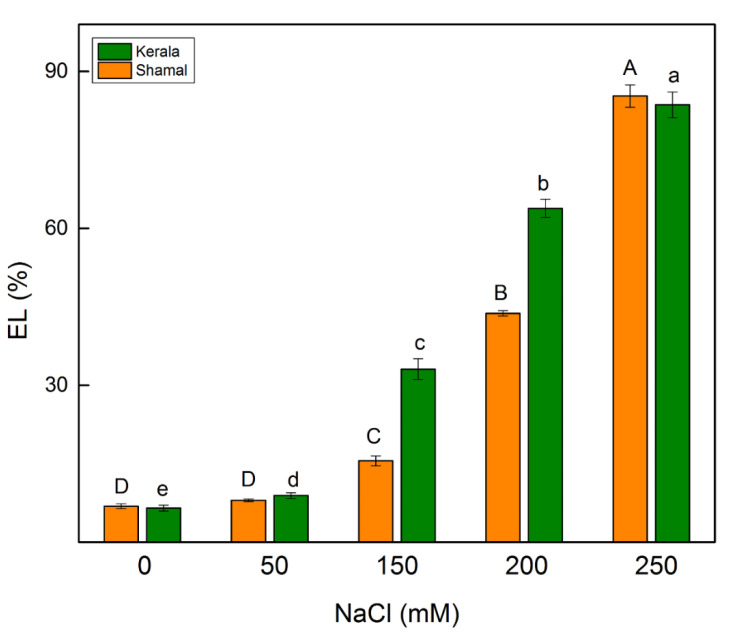
Effects of different NaCl concentrations on the electrolyte leakage (EL) in leaves of sorghum (*Sorghum bicolor* L. Shamal) and maize (*Zea mays* L. Kerala). Mean values (± SE) were calculated from 8 independent measurements. Different letters indicate significant differences for different NaCl concentrations at *p* < 0.05 (uppercase for sorghum and lowercase for maize).

Electrolyte leakage (EL) from plant tissues results from alteration of the cell membrane integrity and is used as a fast indicator when identifying salt tolerance [38]. A statistically significant increase in the EL value was registered at the lowest NaCl concentration (50 mM NaCl) only in maize. Significant differences in sorghum and maize regarding EL values were found after treatment with 150 mM and 200 mM NaCl, as the increases were greater in maize (by 33–64%) than in sorghum (by 16–44%) (Figure 2).

### 2.4. Room Temperature Chlorophyll a Fluorescence

**Figure 3:**
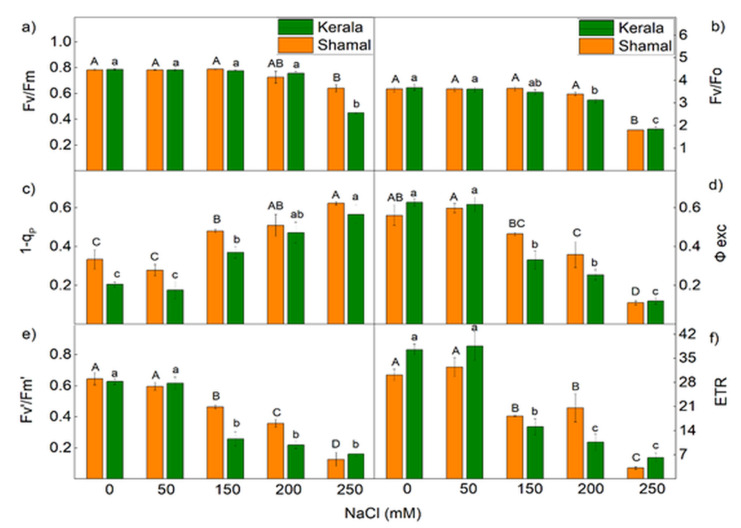
Effects of different NaCl concentrations on the PAM parameters of the chlorophyll fluorescence in leaves of sorghum (*Sorghum bicolor* L. Shamal) and maize (*Zea mays* L., Kerala): the maximum quantum efficiency of PSII in the dark-adapted state, Fv/Fm (**a**); the maximum ratio of quantum yields of photochemical and concurrent non-photochemical processes in PSII, Fv/Fo (**b**); the amount of closed PSII centers, 1-q_P_ (**c**); the excitation efficiency of open PSII centers, Φ_exc_ (**d**); the effective quantum yield of the PSII photochemistry, Fv’/Fm’ (**e**); the linear electron transport rate, ETR (**f**). Mean values (± SE) were calculated from 8 independent measurements. Different letters indicate significant differences for the respective parameters at *p* < 0.05 (uppercase for sorghum and lowercase for maize).

**Figure 4:**
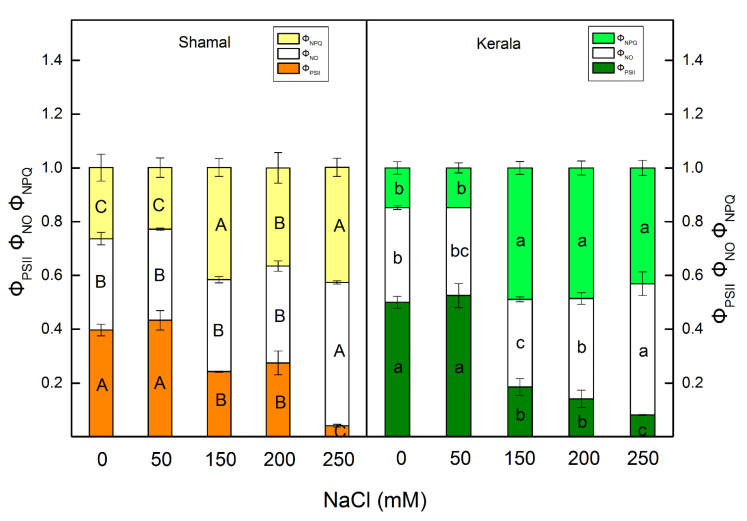
Effects of different NaCl concentrations on the PAM parameters of the chlorophyll fluorescence in leaves of sorghum (*Sorghum bicolor* L. Shamal) and maize (*Zea mays* L. Kerala). The effective quantum yield of photochemical energy conversion of PSII, Φ_PSII_; the ratios of non-regulated (Φ_NO_) and regulated (Φ_NPQ_) energy loss in PSII. Mean values (± SE) were calculated from 8 independent measurements. Different letters indicate significant differences for the respective parameters at *p* < 0.05 (uppercase for sorghum and lowercase for maize).

The effects of salinity on the parameters of PAM chlorophyll fluorescence in both studied species varied depending on the applied NaCl concentrations (Figure 3a–f and Figure 4). The maximum quantum yields of primary PSII photochemistry in the dark-adapted state (Fv/Fm) and the maximum ratio of quantum yields of photochemical and concurrent non-photochemical processes in PSII (Fv/Fo) were influenced only at the highest NaCl concentration (250 mM) in both species, while the effective quantum yield of PSII photochemistry (Fv’/Fm’) was influenced at 150 mM NaCl. Strong influences on the parameters 1-q_P_, ETR and Φ_exc_ were also observed (Figure 3c,d,f). The closed PSII reaction centers were increased by 44% in sorghum and by 80% in maize after treatment with 150 mM NaCl, while after application of 250 mM NaCl these increased by 86% and 175% for sorghum and maize, respectively. In the same concentration range (150–250 mM), the efficiency of the open PSII reaction centers was decreased from 17% to 80% in sorghum and from 47% to 80% in maize, while ETR was decreased from 40% to 90% in sorghum and from 60% to 84% in maize. The salt-induced changes in the parameters ETR, 1-q_P_ and Φ_exc_ were also more pronounced in maize than in sorghum for 150 mM and 200 mM NaCl concentrations.

The higher NaCl concentrations (150–250 mM) influenced the effective quantum yield of the photochemical energy conversion of PSII (Φ_PSII_) and the quantum yields of regulated (Φ_NPQ_) and non-regulated (Φ_NO_) energy losses in PSII (Figure 4). Among untreated plants, the Φ_PSII_ values were slightly higher in maize than in sorghum. Comparing sorghum and maize, significantly different salt-induced changes in the parameter Φ_PSII_ were observed at 150 mM and 200 mM NaCl. At these concentrations, the parameter Φ_PSII_ decreased by 40% in sorghum and by 60–72% in maize. The smaller Φ_PSII_ value correlated with an increase mainly in the regulated non-photochemical energy loss (Φ_NPQ_). Both regulated (Φ_NPQ_) and non-regulated (Φ_NO_) energy losses in maize and sorghum tended to elevate at 150 mM NaCl and the higher concentrations (Figure 4).

**Figure 5:**
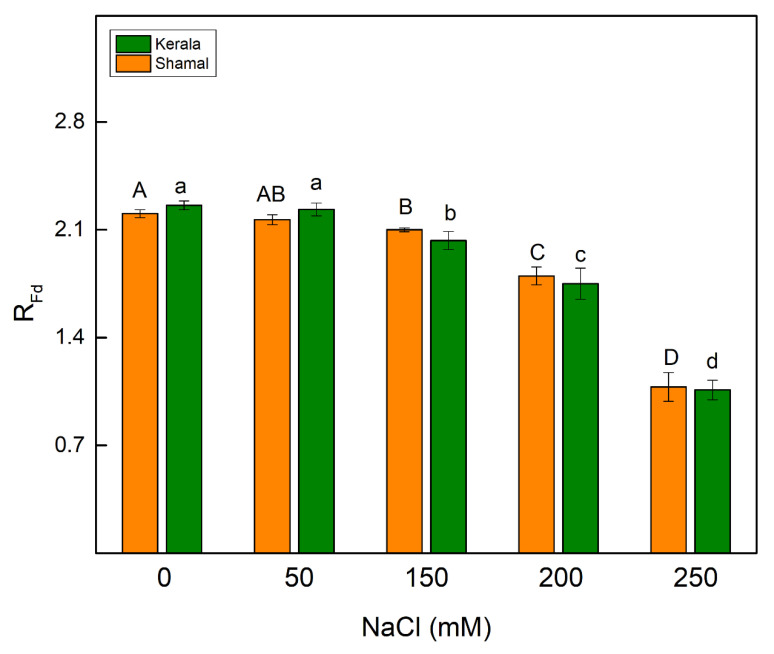
Effects of different NaCl concentrations on the chlorophyll fluorescence decay ratio, R_Fd_ (which corresponds to the rate of photosynthesis), in leaves of sorghum (*Sorghum bicolor* L. Shamal) and maize (*Zea mays* L. Kerala). Mean values (± SE) were calculated from 8 independent measurements. Different letters indicate significant differences for different NaCl concentrations at *p* < 0.05 (uppercase for sorghum and lowercase for maize).

The salt-induced changes in thylakoid membranes inhibited the photosynthetic rate (i.e., parameter R_Fd_ decreased) at concentrations of 150 mM NaCl and above in both studied species (Figure 5).

**Figure 6:**
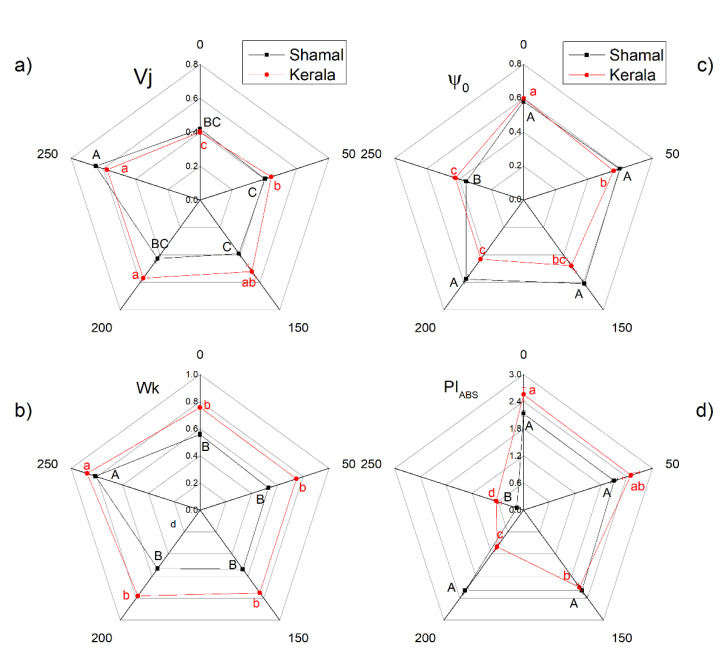
Effects of different NaCl concentrations on certain OJIP parameters in leaves of sorghum (*Sorghum bicolor* L. Shamal) and maize (*Zea mays* L. Kerala): Vj—relative variable fluorescence at 2 ms (**a**); Wk—ratio of the K phase to the J phase, which indicates the donation of electrons by the OEC (**b**); ψo—probability that a trapped exciton transfers as an electron into the electron transport chain beyond Q_A_ (**c**); PI_ABS_—performance index of PSII energy conservation from photons absorbed by PSII to the reduction of intersystem electron acceptors (**d**). Mean values (±SE) were calculated from 8 independent measurements. Different letters indicate significant differences between different NaCl concentrations for the respective parameters at *p* < 0.05 (uppercase for sorghum and lowercase for maize).

The influences of different concentrations of NaCl (50–250 mM) on the selected OJIP parameters in maize and sorghum leaves are shown in Table 2 and Figure 6a–d. Chlorophyll induction curves are fast and reliable indicators of the influence of the abiotic stressors on the photosynthetic light reactions [39,40,41]. The analysis of OJIP parameters revealed that the absorbed light energy per active reaction center (ABC/RC) was influenced in both studied species after treatment with all applied NaCl concentrations, meaning salt-induced changes in apparent antenna size (Table 2). The electron transport flux from Q_A_ to Q_B_ per reaction center (ETo/RC) was also reduced by salinity at higher NaCl concentrations, although to a lesser extent in sorghum (by 4% and 15% for 150 mM and 200mM NaCl, respectively) than in maize (by 13% and 17% for 150 mM and 200 mM NaCl, respectively). The data also revealed that the flow of electrons passing through PSII and reaching the acceptor side of PSI (REo/RC) at all studied NaCl concentrations decreased more in maize than in sorghum. A decrease of the quantum yield for the reduction of end electron acceptors at the PSI acceptor side (ϕ_R0_) was also registered after NaCl treatment, as the changes in maize were observed at all studied salt concentrations, while in sorghum they were observed above 150 mM NaCl (Table 2).

At the same time, the performance index on the absorption base (PI_ABS_) was influenced only at 250 mM NaCl in sorghum, while in maize this occurred at all studied concentrations (Figure 6d). The influence of salinity on the parameters (Vj and ψo) showed that the activity levels of the acceptor side of PSII were very different in both studied species (Figure 6a,c). Changes in the probability of the trapped electron to be transferred to an electron transport chain beyond Q_A_ (ψo) and the relative variable fluorescence at 2 ms (Vj) were registered at all salt concentrations in maize, while in sorghum this occurred only at 250 mM NaCl. At the same time, increases in the Wk parameter (the ratio of the K phase to the J phase) at 250 mM NaCl were registered in both studied species, which were related to the activity of the oxygen-evolving complex (OEC) (Figure 6b) [40].

### 2.5. Photooxidation of P_700_

Photooxidation of P_700_ (P_700_^+^) by far-red light was used to quantify the photochemistry of PSI. The far-red light induced absorbance changes at 830 nm (ΔA), which were different in sorghum and maize depending on the NaCl concentration (Table 3). For maize, the ΔA/A ratio decreased significantly after the treatments with 200 mM NaCl (by 27%) and 250 mM NaCl (by 45%), while for sorghum a statistically significant decrease (37%) was observed only at the highest applied (250 mM) NaCl concentration.

The dark reductions of P_700_^+^ in all studied variants of maize and sorghum were deconvoluted into two exponential components (fast and slow) with rate constants k_1_ and k_2_ for the fast and the slow exponents, respectively. In sorghum, the constant k_1_ increased after treatment with 150 mM NaCl (by 60%) and higher NaCl concentrations (Table 3). The opposite effects were observed in maize, whereby the NaCl treatments at concentrations above 150 mM led to decreases of the rate constant k_1_ from 10% to 33%. It was also observed that the different NaCl treatments influenced constant k_2_ in both studied species (maize and sorghum), as the effects strongly depended on NaCl concentrations. The constant k_2_ was influenced at 250 mM NaCl in sorghum and at 150 mM NaCl and higher NaCl concentrations in maize.

## 3. Discussion

The impacts of salt stress on physiological and biochemical processes in plant species, including the functions of their photosynthetic apparatus, vary depending on salt concentrations, the duration of stress, and the plant’s genotype [4,8,10,15,36]. It has been shown that salt stress decreases the pigment amount, depending on the tolerance of the plant species. Moreover, in some salt-tolerant species, the pigment content increases [5]. Our results reveled reductions of total Chl content in both studied species (Table 1), although the effect was more pronounced in maize than in sorghum. Similar reductions in Chl content in maize and sorghum under salt stress were also observed in previous studies [34,42,43,44]. The salt-induced influence on Chl content could be due to impaired biosynthesis or accelerated chlorophyll degradation [45]. Carotenoids, which are important for the photoprotection of plants, were reduced after salt exposure in maize (at 50 mM NaCl and above) and sorghum (above 150 mM NaCl) (Table 1). The variations in Chl and Car contents after treatment with different NaCl concentrations also influenced the Chl *a*/*b* and Car/Chl ratios. The increases of the Chl *a*/*b* ratio under salinity in the studied plant species suggested decreases of the light-harvesting complex of PSII [46,47] and a number of the granal thylakoids [47,48]. These results are in accordance with electron microscopic studies showing that increased salinity leads to a disintegration of the granal thylakoids and damage to stromal thylakoids [18,49]. It was been shown that genes encoding the proteins of light-harvesting complexes (Lhca1 and Lhcb1-5) in sorghum were downregulated [50]; therefore, it could be suggested that the more reduced Chl *a*/*b* ratio in sorghum than in maize after salt stress caused structural reorganization in the main pigment–protein complexes and unstacking of the membranes, which in turn may have enhanced their tolerance to salt stress [26].

Salinity enhances reactive oxygen species (ROS) in plants, which leads to oxidative stress, causing lipid peroxidation and disruption of the membrane integrity [13,44,51]. ROS directly promote PSII damage or inhibit repair of PSII [52,53]. The treatment with NaCl (150 mM and higher) strongly increased the H_2_O_2_ contents in maize and sorghum (Figure 1), although the increase in H_2_O_2_ was bigger in maize (63% and 72% for plants treated with 150 mM NaCl and 200 mM NaCl, respectively) than in sorghum (24% and 35% for plants treated with 150 mM NaCl and 200 mM NaCl, respectively). Previous investigations have shown the expression of ROS-associated antioxidant enzymes in sorghum induced by salt stress, which can be used to adapt the plants to salinity [54,55].

The enhanced H_2_O_2_ amount at higher NaCl concentrations (150 mM and 200 mM) led to increases in lipid peroxidation (estimated as MDA content) of 24–35% in sorghum and 63–74% in maize (Figure 1). Moreover, the data revealed that the electrolyte leakage increased in both studied species at higher NaCl concentrations, as the changes were more pronounced in maize (33% for 150 mM and 64% for 200 mM NaCl) than in sorghum (16% for 150 mM and 84% for 200 mM NaCl) (Figure 2), suggesting disruption of the membrane integrity after salt treatment. These results led us to infer that the studied species have different levels of salt tolerance. The better salt tolerance of sorghum than maize could be due to a stronger increase in the content of osmoregulation solutes in sorghum under salt stress [55,56,57].

Changes in pigment content and membrane injury under salt stress caused a decrease of the efficiency of the photosynthetic machinery. A quick indicator for impacts of salinity on the functions of the photosynthetic apparatus is chlorophyll *a* fluorescence [58,59,60,61]. In our experiments, we used chlorophyll fluorescence measurements (PAM and OJIP test) to estimate the impacts of salinity on the photosynthetic apparatus. The potential PSII efficiency of dark-adapted leaves was estimated using the Fv/Fm ratio and the more sensitive Fv/Fo ratio [62]. The maximal efficiency of PSII (Fv/Fm) was reduced only at the highest studied NaCl concentration (250 mM), indicating PSII inhibition induced by salinity. Significant differences in the maximum ratios of quantum yields of photochemical and concurrent non-photochemical processes in PSII (Fv/Fo) were observed after treatment with 150 mM NaCl and higher concentrations in maize and 200 mM and 250 mM NaCl in sorghum (Figure 3a,b). Salt-induced decreases in Fv/Fo ratio suggest structural damage of the thylakoid membranes [63]. Higher NaCl concentrations (above 150 mM NaCl) increased the closed PSII centers (1-q_P_), decreased Φ_PSII_ and inhibited the ETR in maize more strongly than in sorghum (Figure 3c and Figure 4). The data in the present study also revealed the influence of salinity on the electron flux from Q_A_ to plastoquinone (ETo/RC) (Table 2). The decreases of the electron flux in maize occurred at all studied NaCl concentrations, while in sorghum this process was influenced only at 200 mM and 250 mM NaCl. A decrease of photochemical quenching as a result from a restriction of the electron flow from Q_A_^−^ to plastoquinone pool under salt stress was shown in our previous investigation with *Paulownia* [27,64]. It has also been found that higher salinity influences Q_A_^−^ reoxidation by plastoquinone and by the recombination of electrons in Q_A_Q_B_^−^ via the Q_A_^−^Q_B_ ↔ Q_A_Q_B_^−^ charge equilibrium. Additionally, lower efficiency of the open PSII centers after the salt treatment (Φ_exc_) was observed, with the effect being stronger in maize than in sorghum (Figure 3d). Our data also revealed that decreases of Φ_PSII_ correspond to increases of the quantum yields of regulated (Φ_NPQ_) and non-regulated (Φ_NO_) energy losses in PSII (Figure 4). It should be noted that the increases of Φ_NO_ were similar in both studied species, while the changes in Φ_NPQ_ were much higher in maize (290–340%) than in sorghum (135–159%). Furthermore, our data revealed changes in acceptor (parameters Vj and ψo) and donor (parameter Wk) sides of PSII (Figure 6a–c). The Wk value corresponds to the activity of the OEC, while an increase of Wk indicates dissociation of this complex and damage [57]. A recent study revealed the correlation between salt-resistant sorghum genotypes and changes in this parameter [65]. The impacts of salinity on both sides of PSII were observed in maize at all studied concentrations, while in sorghum this occurred only at the highest studied concentration. The similar impacts of salinity on both sides of PSII have also been shown in wheat [24]. Salt-induced changes in PSII photochemistry resulted in decreases of the efficiency index on the absorption base, PI_AB_s (Figure 6d), and the rate of photosynthesis, R_Fd_ (Figure 5), at higher salt concentrations.

In sorghum, the influence on the P_700_ photooxidation was observed only at the highest NaCl concentration (250 mM), while in maize this occurred at 200 mM and 250 mM NaCl (Table 3). Data demonstrated that the constants of the dark relaxation of P_700_^+^ (k_1_ and k_2_) were affected differently depending on the applied NaCl concentration. The constants k_1_ and k_2_ characterized two different electron donor systems or two different pools of PSI located in different domains of thylakoid membranes [66]. The higher NaCl concentrations (above 150 mM) led to increases of k_1_ in sorghum and decreases in maize (Table 3). This indicates that under salt stress, the cyclic electron flow (CEF) around PSI increases in sorghum, which plays a significant role in preventing oxidative damage of the photosynthetic apparatus under stress factors [67,68,69]; however, the increase of CEF in maize was not registered. In addition, changes were also observed in k_2_ in both studied species. The above data suggest that salinity influences both PSI populations (in stroma lamellae and grana margin), although in different ways in sorghum and maize. The alterations in the PSI activity under salt stress for both studied plant species may have been associated with the lowering of electron transport from Q_A_ to the PSI end electron acceptors (REo/RC) and the probability of their reduction (ϕ_Ro_) (Table 2).

## 4. Materials and Methods

### 4.1. Plant Growth Conditions and Treatments

The seedlings from new hybrid lines of maize (*Zea mays* L. Kerala) and sorghum (*Sorghum bicolor* L. Shamal) were used in this study. The seeds were offered by Euralis Ltd. (Lescar, France), one of the big companies that creates cereal hybrids for Europe. After germination, the seedlings were grown in half-strength Hoagland solutions containing: 2.5 mM KNO_3_, 2.5 mM Ca(NO_3_)_2_, 1 mM MgSO_4_, 0.5 mM NH_4_NO_3_, 0.5 mM K_2_HPO_4_, 23 μM H_3_BO_3_, 4.5 μM MnCl_2_, 0.4 μM ZnSO_4_, 0.2 μM CuSO_4_, 0.25 μM Na_2_MoO_4_ and 20 μM Fe-EDTA (pH 6.0). The cultivation of the seedlings was carried out in a photothermostat with controlled conditions: a 12 h light/dark photoperiod, a light intensity of 150 μmol/m^2^s, 28 °C (daily)/25 °C (night) temperature and 60% relative humidity. After 15 days of growth, NaCl was added to final concentrations of 50 mM, 150 mM, 200 mM and 250 mM in nutrient solution for 6 days. The plants that grew without NaCl were used as controls in the study. The electrical conductivities (EC) of nutrient solutions with different NaCl concentrations corresponded to EC of soils with different salinity levels according Ivushkin et al. [2]. The EC of the nutrient solution without and with NaCl were as follows: 0 mM NaCl (1.01 mS/cm, corresponds to non-saline soil), 50 mM NaCl (5.99 mS/cm, corresponds to moderate saline soil), 150 mM NaCl (14.70 mS/cm, corresponds to very saline soil), 200 mM (18.80 mS/cm, corresponds to very saline soil) and 250 mM NaCl (23.20 mS/cm, corresponds to highly saline soil). Two independent experiments (10–15 uniform seedlings for each treatment, about 10 plants in pot) were performed for each treatment. For the analyses, we used the mature leaves (the middle part of the fourth leaf).

### 4.2. Pigment Content

The pigments were extracted from leaves with ice-cold 80% (*v*/*v*) acetone in the dark. The resulting homogenates were centrifuged at 2500× *g* for 8 min at 4 °C. The amounts of total chlorophylls (Chl *a* + *b*) and carotenoids (Car) were determined, as well as the Chl *a*/*b* and Car/Chl ratios. The measurements were made on a spectrophotometer (Specord 210 PLUS, Edition 2010, Analytik-Jena AG, Jena, Germany). The chlorophylls and carotenoids were determined spectrophotometrically at 470 nm, 646.8 nm, and 663.2 nm using the equations of Lichtenthaler [70]:

Chl *a* (μg/mL) = 12.25 A_663.2_−2.79 A_646.8_ (1)

Chl *b* (μg/mL) = 21.50 A_646.8_−5.10 A_663.2_ (2)

Car (μg/ mL) = (1000 A_470_−1.82 Chl *a*−85.02 Chl *b*)/198 (3)

### 4.3. Determination of Oxidative Stress Markers

The content of hydrogen peroxide (H_2_O_2_) was determined using the method proposed by Yotsova et al. [71]. Leaf tissue samples were homogenized with 1% (*w*/*v*) trichloroacetic acid (TCA) at 4 °C then centrifuged at 14,000× *g* for 20 min. The reaction mixture consisted of 0.5 mL leaf extract supernatant, 0.5 mL 100 mM Na-K-phosphate buffer (pH 7.6) and 1 mL 1 M KI reagent (in fresh double-distilled water). The blank probe consisted of 1% TCA with no leaf extract. After 1 h in the dark, the absorbance at 390 nm (Specord 210 Plus, Edition 2010, Analytik Jena AG, Jena, Germany) was measured. The amount of H_2_O_2_ was calculated with molar extinction coefficient at 0.28 μM/cm and expressed as nmol per g dry weight (DW).

The level of lipid peroxidation was determined by estimating malondialdehyde (MDA), which is a decomposition product of peroxidized polyunsaturated fatty acid components of membrane lipids. The MDA content was determined as previously described by Yotsova et al. [71]. The leaf tissues (100 mg) were homogenized with 1% (*w*/*v*) trichloroacetic acid (TCA) followed by centrifugation at 14,000× *g* for 20 min. The reaction mixture consisted of 0.5 mL 1% (*w*/*v*) TCA leaf extract supernatant, 0.5 mL 100 mM Na-K-phosphate buffer (pH 7.6) and 1 mL reagent (20% (*w*/*v*) TCA containing 0.5% (*w*/*v*) TBA. The mixture was heated at 95 °C for 30 min and then cooled to stop the reaction. The tubes were centrifuged at 12,000× *g* for 5 min. The absorbance was measured at 532 nm (Specord 210 Plus, Edition 2010, Analytik Jena AG, Jena, Germany). The amount of MDA was determined by the molar extinction coefficient (0.155 μM/cm) and expressed as nmol per g DW.

Leaf samples for measurements of H_2_O_2_ and MDA were taken from 10 plants for each variant.

### 4.4. Electrolyte Leakage

The electrolyte leakage was determined from pieces (about 4 cm^2^) of mature leaves from different selected plants. The pieces were immersed in 40 mL tubes with distilled water at room temperature in the dark. After 24 h of incubation, the electrical conductivity (EC1) of the solutions was measured with a conductometer (Hydromat LM302, Neunkirchen am Brand, Germany). Thereafter, the samples were boiled for 30 min and then cooled to room temperature to measure the final electrical conductivity (EC2). The electrolyte leakage (EL) was estimated using the equation: EL (%) = (EC1/EC2) × 100.

### 4.5. Room-Temperature Chlorophyll Fluorescence

The pulse-amplitude-modulated (PAM) chlorophyll *a* fluorescence was measured using a PAM fluorometer (model 101/103, Walz GmbH, Effeltrich, Germany) as described by Stefanov et al. [27]. The dark adaptation of leaves took 20 min. The maximum fluorescence levels in the dark-adapted (Fm) and light-adapted (Fm’) states were determined by 0.8 s saturated pulses at 2800 photon μmol/m^2^ s, which were provided by a Schott KL 1500 lamp (Schott Glaswerke, Mainz, Germany). The actinic light was 150 μmol photons/m^2^ s. The following parameters were determined: the maximum quantum efficiency of PSII in dark-adapted state, Fv/Fm = (Fm−Fo)Fm; the amount of the closed PSII centers, 1-q_P_; the effective quantum yield of photochemical energy conversion of PSII, Φ_PSII_ = (Fm’−Fs)/Fm’; the linear electron transport rate, ETR = Φ_PSII_ × 150 × 0.5; the ratios of non-regulated (Φ_NO_ = F_s_/Fm) and regulated (Φ_NPQ_ = Fs/Fm’−Fs/Fm) energy loss in PSII; the effective quantum yield of PSII photochemistry, Fv’/Fm’ = (Fm’−Fo’)/Fm’; the maximum ratio of quantum yields of photochemical and concurrent non-photochemical processes in PSII, Fv/Fo = (Fm−Fo)/Fo; the excitation efficiency of open PSII centers, Φ_exc_ = Φ_PSII_/q_P_ [72,73].

The chlorophyll fluorescence decay ratio (R_Fd_ = F_d_/F_s_) was determined according to Lichtenthaler et al. [74], where Fd is the fluorescence decrease from Fm to a steady state chlorophyll fluorescence (Fs) after continuous saturated illumination (2800 μmol photon/m^2^ s). This ratio (R_Fd_) correlates with the net assimilation of CO_2_ [74].

Chlorophyll *a* fluorescence transitions were measured with a Handy PEA+ instrument (Hansatech, Norfolk, UK). The measurements were taken after dark adaptation for 15 min using leaf clips. Prompt chlorophyll fluorescence was measured by inducing a strong light pulse on dark-adapted leaves. The light pulse intensity was 3000 μmoles/m^2^ s. These measurements were repeated five times. All leaves showed multiphase chlorophyll fluorescence increases during the first second of illumination after dark adaptation. The measured data were used for calculation of the following JIP test parameters [40,75,76]: ABS/RC—specific absorption flux per reaction center (RC), i.e., effective antenna size of an active RC; ETo/RC—electron transport flux per RC further than Q_A_; REo/RC—electron flux per active RC reducing the end electron acceptors on the acceptor side of PSI (at t = 0); ϕ_Po_—maximum quantum yield of primary PSII photochemistry (at t = 0); ϕ_Ro_—quantum yield for reduction of end electron acceptors at the PSI acceptor side; PI_ABS_—performance index of PSII energy conservation from photons absorbed by PSII to the reduction of intersystem electron acceptors; Vj—relative variable fluorescence at 2 ms; ψo—probability that a trapped exciton transfers as an electron into the electron transport chain beyond Q_A_; Wk—ratio of the K phase to the J phase, which indicates the donation of electrons by the oxygen-evolving complex (OEC) [40].

All fluorescence measurements were performed on mature leaves (the middle part of the fourth leaf).

### 4.6. P_700_ Redox State Measurements

The photooxidation of P_700_ (P_700_^+^) was measured on leaves with a dual-wavelength (820 nm) unit (Walz ED 700DW-E) attached to a PAM101E main control unit in the reflectance mode, as described by Dankov et al. [77]. The dark-adapted (20 min) leaves were illuminated with far-red light supplied by a photodiode (102-FR, Walz GmbH, Effeltrich, Germany). Changes in the oxidation of P_700_ (P_700_^+^) were assessed by far-red light-induced changes in absorbance at 820 nm (∆A). To evaluate the effects of NaCl on the photooxidation of P_700_, the ∆A/A ratio was calculated.

### 4.7. Statistical Analysis

Mean values ± SE were calculated from the data for at least two independent treatments with four biological replicates (four plants) of each variant. Statistically significant differences between the effects of different concentrations of NaCl on the studied parameters were identified using one-way ANOVA followed by Tukey’s post hoc tests for each parameter. Prior to the tests, the assumptions of the normality of raw data (using Shapiro–Wilk tests) and the homogeneity of the variance (using Levene’s test) were checked. The homogeneity of variance test was used to verify the parametric distribution of data. Values (± SD) were considered statistically different with *p* < 0.05 after Fisher’s least significant difference post hoc test using Origin 9.0 software (OriginLab, Northampton, MA, USA).

## 5. Conclusions

The variations in the effects of different NaCl concentrations in maize and sorghum suggest differences in salt tolerance levels for these two crop species. The differences between the studied plants were registered mainly at 150 mM and 200 mM NaCl, corresponding to very saline soils, while at moderate and high salinity they showed similar sensitivity. At these concentrations, a stronger influence on the primary PSII photochemistry was observed in maize than in sorghum, corresponding to a decrease of the amount of closed PSII centers (1-q_P_), restriction of electron flow from Q_A_^−^ to plastoquinone pool (ETo/RC) and effective quantum yield of PSII (Φ_PSII_). Inhibition of PSII activity corresponded to increases of the quantum yields of regulated (Φ_NPQ_) and non-regulated (Φ_NO_) energy losses in PSII. Our results suggested that some of the reasons for the better tolerance of the sorghum than maize were the structural alterations in the photosynthetic membranes and the stimulation of the CET around PSI under salt stress. It could be proposed that the smaller H_2_O_2_ amount in sorghum than in maize after NaCl treatment was a result of the better efficiency of its antioxidant system. We cannot exclude the role of osmoregulation solutes, which protect the membrane functions (the amount of MDA was less in sorghum compared to maize after salt treatment), including thylakoid membranes. These results may lead to new applications for sorghum in saline soil and for salt-induced changes in primary PSII photochemistry to assess salt tolerance. Future detailed studies of the antioxidant system, osmoregulatory substances and primary photochemistry of PSII will reveal the exact reasons for the different tolerance levels of these plants.

## Figures and Tables

**Table 1:** Effects of different NaCl concentrations on the pigment contents in leaves of sorghum (*Sorghum bicolor* L. Shamal) and maize (*Zea mays* L. Kerala). Data are given per dry weight (DW). Mean values (±SE) were calculated from 8 independent measurements.

Variant	Chl *a* + *b* (µg/g DW)	Car (µg/g DW)	Chl *a*/*b*	Car/Chl
Shamal				
Control 50 mM NaCl 150 mM NaCl 200 mM NaCl 250 mM NaCl	26017 ± 640 ^A^ 25124 ± 602 ^A^ 18739 ± 924 ^B^ 13694 ± 285 ^C^ 10814 ± 261 ^D^	4979 ± 111 ^A^ 4860 ± 55 ^A^ 4091 ± 273 ^B^ 2978 ± 125 ^C^ 2488 ± 72 ^D^	4.560 ± 0.035 ^D^ 4.602 ± 0.023 ^D^ 4.740 ± 0.010 ^C^ 4.926 ± 0.020 ^B^ 5.293 ± 0.055 ^A^	0.191 ± 0.002 ^B^ 0.193 ±0.003 ^B^ 0.223 ± 0.003 ^A^ 0.217 ± 0.007 ^A^ 0.230 ± 0.009 ^A^
Kerala				
Control 50 mM NaCl 150 mM NaCl 200 mM NaCl 250 mM NaCl	45498 ± 356 ^a^ 39912 ± 340 ^b^ 28860 ± 540 ^c^ 19048 ± 350 ^d^ 13152 ± 141 ^e^	8907 ± 96 ^a^ 7904 ± 27 ^b^ 6174 ± 63 ^c^ 4987 ± 16 ^d^ 4176 ± 28 ^e^	4.449 ± 0.002 ^e^ 4.511 ± 0.007 ^d^ 4.624 ± 0.006 ^c^ 4.741 ± 0.003 ^b^ 4.908 ± 0.004 ^a^	0.197 ±0.002 ^d^ 0.200 ± 0.002 ^d^ 0.214 ± 0.003 ^c^ 0.262 ± 0.001 ^b^ 0.320 ± 0.003 ^a^

Different letters indicate significant differences for the respective parameters at *p* < 0.05 (uppercase for sorghum and lowercase for maize).

**Table 2:** Effects of different NaCl concentrations on the OJIP parameters in leaves of sorghum (*Sorghum bicolor* L. Shamal) and maize (*Zea mays* L. Kerala).

Variant	ABS/RC	ETo/RC	REo/RC	φ_Po_	φ_Ro_
Shamal					
Control 50 mM NaCl 150 mM NaCl 200 mM NaCl 250 mM NaCl	2.821 ± 0.050 ^B^ 2.538 ± 0.047 ^C^ 2.452 ± 0.048 ^C^ 2.419 ± 0.029 ^C^ 4.309 ± 0.165 ^A^	1.297 ± 0.015 ^A^ 1.216 ± 0.013 ^B^ 1.249 ± 0.016 ^A,B^ 1.098 ± 0.020 ^C^ 0.850 ± 0.043 ^D^	0.657 ± 0.052 ^A^ 0.572 ± 0.013 ^A^ 0.513 ± 0.014 ^B^ 0.413 ± 0.014 ^C^ 0.356 ± 0.023 ^C^	0.791 ± 0.001 ^A^ 0.804 ± 0.002 ^A^ 0.794 ± 0.003 ^A^ 0.802 ± 0.003 ^A^ 0.418 ± 0.048 ^B^	0.233 ± 0.022 ^A^ 0.225 ± 0.001 ^A^ 0.209 ± 0.004 ^A^ 0.171 ± 0.004 ^B^ 0.066 ± 0.010 ^C^
Kerala					
Control 50 mM NaCl 150 mM NaCl 200 mM NaCl 250 mM NaCl	2.569 ± 0.050 ^b^ 2.304 ± 0.039 ^c^ 2.226 ± 0.073 ^c^ 2.507 ± 0.055 ^b^ 2.996 ± 0.067 ^a^	1.204 ± 0. 022 ^a^ 1.113 ± 0.018 ^b^ 1.051 ± 0.043 ^b^ 1.001 ± 0.068 ^b,c^ 0.835 ± 0.025 ^c^	0.507 ± 0.020 ^a^ 0.384 ± 0.018 ^b^ 0.339 ± 0.037 ^b,c^ 0.336 ± 0.060 ^b,c^ 0.245 ± 0.043 ^c^	0.796 ± 0.003 ^b^ 0.817 ± 0.004 ^a^ 0.809 ± 0.018 ^a,b^ 0.752 ± 0.008 ^c^ 0.760 ± 0.003 ^c^	0.197 ± 0.013 ^a^ 0.169 ± 0.009 ^a,b^ 0.145 ± 0.011 ^b,c^ 0.120 ± 0.013 ^c,d^ 0.091 ± 0.015 ^d^

ABS/RC—specific absorption flux per reaction center (RC), i.e., effective antenna size of an active RC; ETo/RC—electron transport flux per RC further Q_A_; REo/RC—electron flux per active RC reducing the end electron acceptors on the acceptor side of PSI (at t = 0); φ_Po_—maximum quantum yield of primary PSII photochemistry (at t = 0); φ_Ro_—quantum yield for reduction of end electron acceptors at the PSI acceptor side. Mean values (± SE) were calculated from 8 independent measurements. Different letters indicate significant differences for the respective parameters at *p* < 0.05 (uppercase for sorghum and lowercase for maize).

**Table 3:** Effects of different NaCl concentrations on the photooxidation of P_700_ in leaves of sorghum (*Sorghum bicolor* L. Shamal) and maize (*Zea mays* L. Kerala). The far-red-light-induced steady-state oxidation of P_700_ (ΔA/A) and the fast and slow rate constants (k_1_ and k_2_, respectively) of the P_700_^+^ dark reduction in leaves are presented.

Variant	k_1_	k_2_	ΔA/A
Shamal			
Control 50 mM NaCl 150 mM NaCl 200 mM NaCl 250 mM NaCl	0.357 ± 0.033 ^B^ 0.376 ± 0.041 ^B^ 0.572 ± 0.065 ^A^ 0.502 ± 0.027 ^A^ 0.512 ± 0.038 ^A^	0.046 ± 0.005 ^A,B^ 0.058 ± 0.004 ^A^ 0.040 ± 0.003 ^B^ 0.045 ± 0.004 ^A,B^ 0.022 ± 0.001 ^C^	12.3 ± 0.6 ^A^ 12.7 ± 0.8 ^A^ 11.8 ± 0.2 ^A^ 11.5 ± 0.4 ^A^ 7.8 ± 0.8 ^B^
Kerala			
Control 50 mM NaCl 150 mM NaCl 200 mM NaCl 250 mM NaCl	0.349 ± 0.036 ^a,b^ 0.395 ± 0.032 ^a^ 0.312 ± 0.041 ^a,b^ 0.261 ± 0.027 ^b^ 0.235 ± 0.026 ^b^	0.039 ± 0.004 ^a,b^ 0.045 ± 0.005 ^a^ 0.032 ± 0.002 ^b,c^ 0.024 ± 0.004 ^c^ 0.028 ± 0.002 ^b,c^	12.8 ± 0.5 ^a^ 11.5 ± 0.4 ^a^ 11.6 ± 0.5 ^a^ 9.3 ± 0.5 ^b^ 7.1 ± 0.6 ^c^

Mean values (± SE) were calculated from 8 independent measurements. Different letters indicate significant differences between different NaCl concentrations for the respective parameters at *p* < 0.05 (uppercase for sorghum and lowercase for maize).

## Data Availability

Not applicable.
